# ICSI/IVF treatments allocation using CASAs compared to manual semen analyses

**DOI:** 10.1097/MD.0000000000041501

**Published:** 2025-02-07

**Authors:** Murong Xu, Mingpeng Zhao, Huixia Yang, Minqi Liu, Carol Pui Shan Chan, Ka Kei Fung, Jacqueline Pui Wah Chung, Ellis Kin Lam Fok, David Yiu Leung Chan

**Affiliations:** a Assisted Reproductive Technology Unit, Department of Obstetrics and Gynaecology, Faculty of Medicine, The Chinese University of Hong Kong, Hong Kong, China; b School of Biomedical Sciences, Faculty of Medicine, The Chinese University of Hong Kong, Hong Kong, China; c Heal Fertility Limited, Hong Kong, China.

**Keywords:** CASA, ICSI, semen analysis, sperm concentration, sperm morphology, sperm motility

## Abstract

The manual method of semen analysis is vital in andrology laboratories. To solve the labor-intensive, time-consuming, and subjective problem, computer-assisted sperm analysis (CASA) systems have been developed. However, it is unclear whether the consistency of semen analysis results especially in male infertility diagnoses can be achieved. A total of 326 individuals were recruited from January 14, 2020, to October 22, 2020. The manual method was used as the gold standard. Pairwise comparisons were conducted between CASAs (Hamilton-Thorne CEROS II Clinical, LensHooke X1 Pro, and SQA-V Gold Sperm Quality Analyzer) and manual method. LensHooke had the best performance in concentration, while the others showed moderate performances (intraclass correlation coefficient [ICC]: CEROS-0.723, LensHooke-0.842, SQA-V-0.631). CEROS II had moderate performances in motility, and the others only had poor agreements (ICC: CEROS-0.634, LensHooke-0.417, SQA-V-0.451). Morphology analyses were not consistent with manual results (ICC: LensHooke-0.160, SQA-V-0.261). In Bland–Altman plots, CEROS II (*P* = .379) and SQA-V Gold (*P* = .218) showed consistent measurements in concentrations and total sperm count with the manual method, while the others were inconsistent. LensHooke X1 Pro (κ=0.701) and CEROS II (κ = 0.664) showed substantial performances in oligozoospermia, and SQA-V Gold had moderate agreements (κ = 0.588). In asthenozoospermia, LensHooke X1 Pro had moderate performances (κ = 0.405) and CEROS II had fair agreement (κ = 0.249), while low agreement using SQA-V Gold (κ = 0.157). In teratozoospermia, LensHooke X1 Pro (κ = 0.177) and SQA-V Gold (κ = 0.008) could not have consistent results either. When choosing treatment based on morphology, the ratio of intracytoplasmic sperm injection (ICSI) approximates 0.5 in our unit. However, the ratios were around 0.31 and 0.15 using LensHooke X1 Pro and SQA-V Gold, indicating the reduction of ICSI work in routine treatment. CASA results were not consistent with manual results, and the deviations might result in skewed in vitro fertilization/ICSI allocation in subsequent treatment. Interestingly, tested CASA systems tend to skew to conventional in vitro fertilization instead of ICSI. Although CASA technologies have been improving recently, the manual method cannot be replaced by the tested CASA systems at present and the results should be treated with caution. CASA algorithms should be improved, especially in morphology. Future strict studies should be designed to evaluate the CASA systems with both internal and external validations.

## 1. Introduction

Infertility is an important public health issue globally.^[1]^ It is estimated that around 48 million couples experience infertility problems all over the world.^[2]^ Male infertility occupies around 40% to 50% of the infertility issue.^[3,4]^ With the introduction of assisted reproductive technologies (ART), semen analysis (SA) is increasing in demand. SA with high accuracy provides information to both the patients and doctors about male fertility status and assists them to choose a proper treatment.^[5–7]^ Although the results of the manual method need only low cost and are reliable with experienced personnel, they may still suffer from multiple variables and can be time-consuming and subjective as well. Moreover, regular personnel training and quality control are required.^[8–11]^

To solve the problems of manual method, computer-assisted sperm analysis (CASA) systems have been developed.^[12]^ However, relatively few studies compare the consistency between different CASA systems with the manual SA.^[6,13–15]^ The analysis results highly depend on the instrument and settings.^[16]^ Additionally, quality control and standardization of CASA have become another concern since different CASA systems use different algorithms, which makes it hard to compare the results between different systems.^[6,13,17,18]^

Sperm morphology evaluation is an important part of routine SA, but it is labor-intensive, time-consuming, and subjective. In the IVF procedure, sperm morphology is a factor in choosing the treatment between intracytoplasmic sperm injection (ICSI) and conventional in vitro fertilization (IVF).^[19]^ During treatment, ICSI only selects a single sperm to fertilize with the oocyte, which bypasses the penetration with the oocyte and is more complex and costly. Although CASA can be used for morphology evaluation, the generated results are considered to have discrepancies with manual results in many studies.^[5,20–22]^

Manual SA requires much time and effort. High consistency of automatic CASA methods with the manual method has the potential to improve accuracy and efficiency. Application of CASA with high consistency can not only provide reliable results with reduced variations by preventing human errors but also reduce repetitive tasks and optimize resource utilization. The reliable results and shortened operation time can then contribute to fast clinical decision-making and enhance patient experience. In this study, we aimed to compare the SA results of sperm concentration, motility, and morphology using 3 different CASA systems: Hamilton-Thorne CEROS II Clinical, LensHooke™ X1 Pro, and SQA-V Gold™ Sperm quality analyzer. Also, we wanted to evaluate the role of CASA in sperm morphology for choosing IVF treatment. An understanding of their different properties can help technicians utilize the advantages of different systems to better assess sperm parameters by complementing each other in laboratory practice. The evaluation of the performances of different CASA systems is beneficial to understand to what extent can CASA performances replace manual performance, especially for choosing fertility treatment based on sperm morphology.

## 2. Methods

### 2.1. Ethics statement

The study was reviewed and approved by the institutional review board of the Joint Chinese University of Hong Kong – New Territories East Cluster Clinical Research Ethics Committee (CREC No. 2016.499). Participants who were willing to donate the semen samples were included and informed consent was obtained.

### 2.2. Manual method

Manual SA was performed by an experienced andrologist according to the 5th World Health Organization (WHO) laboratory manual for the examination and processing of human semen.^[23]^ Internal quality control is conducted monthly, and the unit is a member of the United Kingdom National External Quality Assessment Service (UK NEQAS). Thus, the manual method was considered the gold standard. Concentration, motility, and morphology were evaluated under the Nikon Eclipse E400 trinocular microscope (Nikon) in duplicates. The concentration of sperm was calculated in the improved Neubauer counting chamber at × 400 magnification. Sperm motility was evaluated at × 400 magnification and classified into progressive motility (PR), nonprogressive motility (NP), and immotility (IM). The diff-quik method was performed to evaluate the morphology at × 1000 oil-immersion magnification (Halotech).

### 2.3. CASA systems

#### 2.3.1. CEROS II clinical sperm analyzer

The CEROS™ II clinical sperm analyzer (Hamilton Thorne), together with an external negative phase contrast trinocular BX43 microscope (Olympus), a portable MiniTherm stage warmer (Hamilton Thorne), was connected to the computer. This system can be applied in multispecies sperm analysis in concentration, motility, and morphology. It can also generate metrics of velocities and morphometry. Three-microliter semen samples were applied on calibrated Leja 4 chambers slides (IMV Technologies) and evaluated after distributing evenly.

#### 2.3.2. LensHooke™ X1 Pro semen quality analyzer

LensHooke™ X1 Pro semen quality analyzer (Bonraybio) quantitatively analyses the concentration, motility, morphology, and pH automatically. Test cassettes have 2 drip areas, 1 for pH and the other for other parameter evaluations, with anti-leakage function to prevent interference from other factors. Dual drips of 40 µL semen were applied on the 2 windows of cassettes for evaluation.

#### 2.3.3. SQA-V Gold™ sperm quality analyzer

The SQA-Vision Gold™ sperm quality analyzer (SQA-V Gold) (medical electronic systems) is an automatic analyzer that can perform SA in 75 seconds. The disposable capillary was filled with >50 μL of the semen samples and pushed the syringe piston into the separating valve for measurement. It can display concentration, motility, morphology, and velocity automatically. Semiautomated parameters like morphology differential, vitality, pinheads, and other cells can also be generated.

### 2.4. Statistical analysis

The analyses were conducted in R (version 4.3.2). *P* < .05 was considered significantly different. The comparisons were pairwise (manual vs a single CASA system).

#### 2.4.1. Comparison of continuous data

Student *t* test was used for continuous variables with normal distribution to test the difference and Wilcoxon signed-rank test was used for continuous variables without normal distribution. The comparisons were 2-sided.

#### 2.4.2. Intraclass correlation coefficient

Intraclass correlation coefficient is a descriptive statistic used to measure the consistency of the units within the same group.^[24]^ IRR package and the 2-way random-effects model were used in this study. Koo and Li gave the guidelines that ICC < 0.5, 0.5 to 0.75, 0.75 to 0.9, and > 0.9 were considered poor, moderate, good, and excellent respectively.^[25]^

#### 2.4.3. Linear regression

Linear regression is used to model relationships between variables. It simply had 1 input and 1 output variable in this study.^[26,27]^ The linear relationship of CASA results and manual results were predicted.

#### 2.4.4. Bland–Altman analysis

Bland–Altman analysis is used to evaluate the correlation and agreement of different laboratory methods. It is the most suitable method to analyze the difference between the gold standard and new methods to determine the feasibility.^[28,29]^ For scatter dots in Bland-Altman plots, locally estimated scatterplot smoothing (LOESS) were used to fit the trend.

#### 2.4.5. Cohen kappa coefficient (κ)

κ is used to evaluate the reliability of categorical data among different collectors. The range is from −1 to +1. Values ≤0, 0.01 to 0.20, 0.21 to 0.40, 0.41to 0.60, 0.61 to 0.80, 0.81 to 1.00 are considered as no, none to slight, fair, moderate, substantial, and almost perfect agreement respectively.^[30]^

#### 2.4.6. Bootstrapping analysis

Bootstrapping was used to evaluate the uncertainty of the regression model.^[31]^ The analysis was performed by resampling with 1000 replicates and the R square statistics were calculated including the 95% confidence intervals (CI) and biases.

## 3. Results

### 3.1. Summarized information of participants and semen parameters

The study was conducted in the ART Unit of the Prince of Wales Hospital from January 14, 2020, to October 22, 2020. We recruited 326 participants aged from 20 to 59, with an abstinence period of 2 to 7 days (Table 1). Two hundred six samples were chosen to use CEROS II. Three hundred twenty three samples were chosen to use LensHooke X1 Pro. 45 samples were chosen to use SQA-V Gold.

**Table 1:** Summarized semen parameter values and reportable ranges of the 3 CASA systems.

Semen parameters	Manual		CEROS II		LensHooke X1 Pro		SQA-V Gold	
	Median (5th centiles–95th centiles)	n	Median (5th centiles–95th centiles)	n	Median (5th centiles–95th centiles)	n	Median (5th centiles–95th centiles)	n
Semen volume (mL)	2.50 (0.90–5.28)	326	–	206	–		–	
Sperm concentration (×10^6^/mL)	32.60 (2.81–129)		31.2 (4.29–133.00)		47.20 (0.75–202)	323	54.3 (7.54–137)	45
Total sperm count (×10^6^)	81.30 (5.62–356)		67.7 (9.73–638.00)		107.00 (2.59–461.00)	323	120.00 (16.50–287.00)	45
Total motility (PR + NP%)	50.00 (23.00–63.00)		26.10 (4.38–65.40)		56.00 (1.00–93.80)	322	48.00 (14.00–66.00)	40
PR (%)	46.00 (18.00–60.00)		24.90 (4.14–64.30)		43.00 (1.00–78.60)	322	32.00 (9.90–51.20)	40
NP (%)	4.00 (2.00–6.00)		0.43 (0.00–2.61)		10.00 (1.00–28.80)	322	11.00 (4.00–25.50)	40
IM (%)	50.00 (37.00–76.9)		73.90 (34.60–95.60)		44.00 (6.20–99.00)	322	52.00 (34.00–86.00)	40
Normal morphology (%)	3.20 (0.80–6.00)		–		5.00 (1.00–9.25)	287	6.00 (3.00–12.10)	39
*Reportable range*								
pH			–		6.0 to 8.0 (each scale range: 0.2)		–	
Concentration			<2 to 300 (10^6^/mL)		<0.1% to 300% (10^6^/mL)		<2 to 400 (10^6^/mL)	
Total motility			0% to 100%		<1% to 100%		0% to 100%	
Morphology			0% to 100%		<1% to 100%		2% to 30%	

This table shows the values of different semen parameters with manual and 3 CASA methods in medians and ranges from the 5th centiles to the 95th centiles. The WHO values were also displayed for reference. The 5th centiles of the WHO reference semen parameter values were considered as the lower reference limits in WHO guidelines. This table also shows the reportable ranges of the 3 CASA systems. Parameters within these ranges were reliable according to the CASA manual.

CASA = computer-assisted sperm analysis, IM = immotility, NP = nonprogressive motility, PR = progressive motility, WHO = World Health Organization.

### 3.2. Total performance

**Figure 1.**
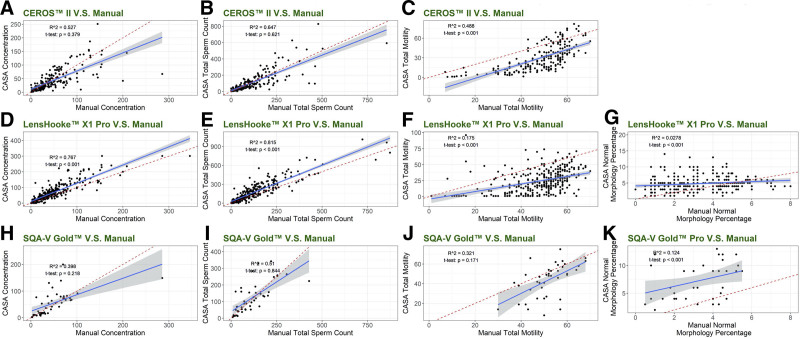
Linear regression analysis. LensHooke X1 Pro performed best in concentration and total sperm count, while CEROS II performed best in motility. However, both performed poor in morphology. Linear regression was used to model the relationship between variables. (A) to (C) show the comparisons between the manual method and CEROS II in sperm concentration, total sperm counts, and total motility. (D–G) and (H–K) show the comparisons between the manual method with LensHooke™ X1 Pro and SQA-V Gold™, respectively, in sperm concentration, total sperm counts, total motility, and normal sperm morphology. The blue lines represented the fitted lines of the relationships between the 2 methods, while the red line represented the line: y = x. Adjusted R square and *P*-values were shown at the upper left.

The results of sperm concentration and total sperm count (Fig. 1A, D, H, and B, E, I) demonstrated that there were significant nonzero correlations existing only between the performance of LensHooke X1 Pro and the manual 1 (*P*-values < .001). ICCs of comparisons of CEROS II, LensHooke X1 Pro, and SQA-V Gold with manual counting were 0.723, 0.842, and 0.631 (Table 2), indicating that LensHooke X1 Pro had the best performance on concentration, and then CEROS II and SQA-V Gold. The analysis also showed that the results of CEROS II and SQA-V Gold were statistically lower than manual ones while those of LensHooke X1 Pro were statistically higher.

**Table 2:** Overall results of the data analysis on comparisons between manual methods and CASA systems.

CEROS II	Concentration	Total sperm counts	Total motility	PR	NP	IM	Morphology
Sample size	206	206	206	206	206	206	
*R* ^2^	0.527	0.647	0.489	0.507	0.003	0.486	
*P*-value	.001	.001	.001	.001	.418	.001	
ICC (95% CI)	0.723 (0.651–0.782)		0.634 (0.545–0.709)				
Bootstrapping *R*^2^ CI	0.361 to 0.647		0.418 to 0.551				
Bootstrapping bias	0.008		0.002				

LensHooke™ X1 Pro	Concentration	Total sperm counts	Total motility	PR	NP	IM	Morphology
Sample size	323	323	322	322	322	322	287
*R* ^2^	0.767	0.815	0.291	0.258	0.001	0.291	0.028
*P*-value	.001	.001	.001	.001	.614	.001	.004
ICC (95% CI)	0.842 (0.807–0.871)		0.417 (0.322–0.503)				0.160 (0.046–0.271)
Bootstrapping *R*^2^ CI	0.702 to 0.820		0.187 to 0.389				0.004 to 0.077
Bootstrapping bias	0.002		−0.0002				0.003

SQA-V Gold	Concentration	Total sperm counts	Total motility	PR	NP	IM	Morphology
Sample size	45	45	40	40	40	40	39
*R* ^2^	0.398	0.510	0.321	0.417	0.009	0.321	0.124
*P*-value	.001	.001	.001	.001	.552	.001	.030
ICC (95%CI)	0.631 (0.417–0.779)		0.451 (0.166–0.666)				0.261 (−0.055 to 0.53)
Bootstrapping *R*^2^ CI	0.190 to 0.546		0.064 to 0.552				0.004 to 0.388
Bootstrapping bias	0.052		0.015				0.020

This table shows the sample sizes, estimates, *R*-squares, and *P*-values of the comparisons between the manual method and the 3 CASA systems on the parameters of concentration, motility, and morphology. ICCs with 95% CIs and bootstrapping *R*^2^ with biases based on 1000 bootstrap replicates are also presented in the table.

CASA = computer-assisted sperm analysis, CIs = confidence intervals, ICCs = intraclass correlation coefficients, IM-immotility, NP-nonprogressive motility, PR = progressive motility.

Total motility was analyzed and results (Fig. 1C, F, J) showed that there were significant nonzero correlations using LensHooke X1 Pro and CEROS II (both *P* < .001), but not SQA-V Gold (*P*-value = .171). ICCs of comparisons of CEROS II, LensHooke X1 Pro, and SQA-V Gold with manual method were 0.634, 0.417, and 0.451 (Table 2), indicating the moderate performance of CEROS II and bad performances of LensHooke X1 Pro and SQA-V Gold. Motility was significantly higher when using CEROS II, while lower when using LensHooke X1 Pro.

The morphology correlations were also analyzed among the manual method, LensHooke X1 Pro, and SQA-V Gold. The results (Fig. 1G and K) demonstrated that there were significant nonzero correlations (*P* < .001). However, ICCs of comparisons of LensHooke X1 Pro and SQA-V Gold with manual counting were 0.160 and 0.261 (Table 2), indicating both poor performances.

**Figure 2.**
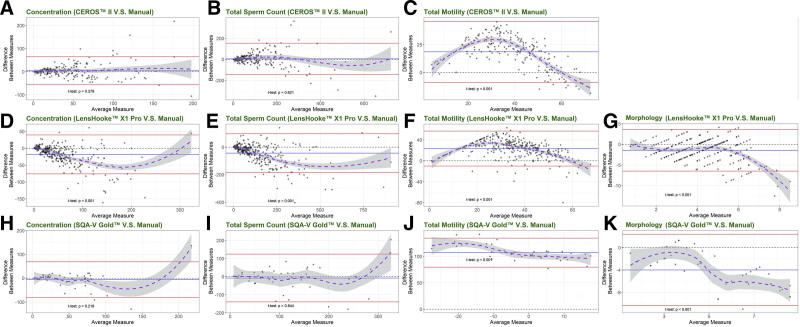
Bland-Altman plots. Differences of concentrations measured by LensHooke X1 and all motility and morphology parameters measured by CASAs were significantly deviated from 0 (*P* < .001). The Bland-Altman analysis was used to evaluate the correlation and agreement of different laboratory methods. (A) to (C) show the differences between the manual method and CEROS II in sperm concentration, total sperm counts, and total motility. (D–G) and (H–K) show the differences between the manual method with LensHooke X1 Pro and SQA-V Gold respectively in sperm concentration, total sperm counts, total motility, and normal sperm morphology in Bland-Altman plots. The blue lines represented the mean of the difference between measures (y-axis) and the red lines represented the 1.96 times standard deviations from means. The dashed line in black indicated the zero difference between measures while the dashed curve in purple was the fitted curve by the LOESS indicating the trends of the data with an area of 95% CI in gray. The longer distance of the dot to the zero indicates the larger difference between the 2 measurements.

Additionally, the Bland-Altman plots displayed the closeness in concentration, total sperm count, motility, and morphology of the 2 measurements (Fig. 2). CEROS II and SQA-V Gold showed no significant differences in concentrations when compared with the manual method, as well as the total sperm count, indicating a high consistency level. However, LensHooke X1 Pro showed significantly different concentrations (*P* < .001). For motility and morphology, the differences between any of the tested CASA systems and the manual method significantly deviated from 0 (all *P* < .001).

### 3.3. Clinical application of CASA

**Figure 3.**
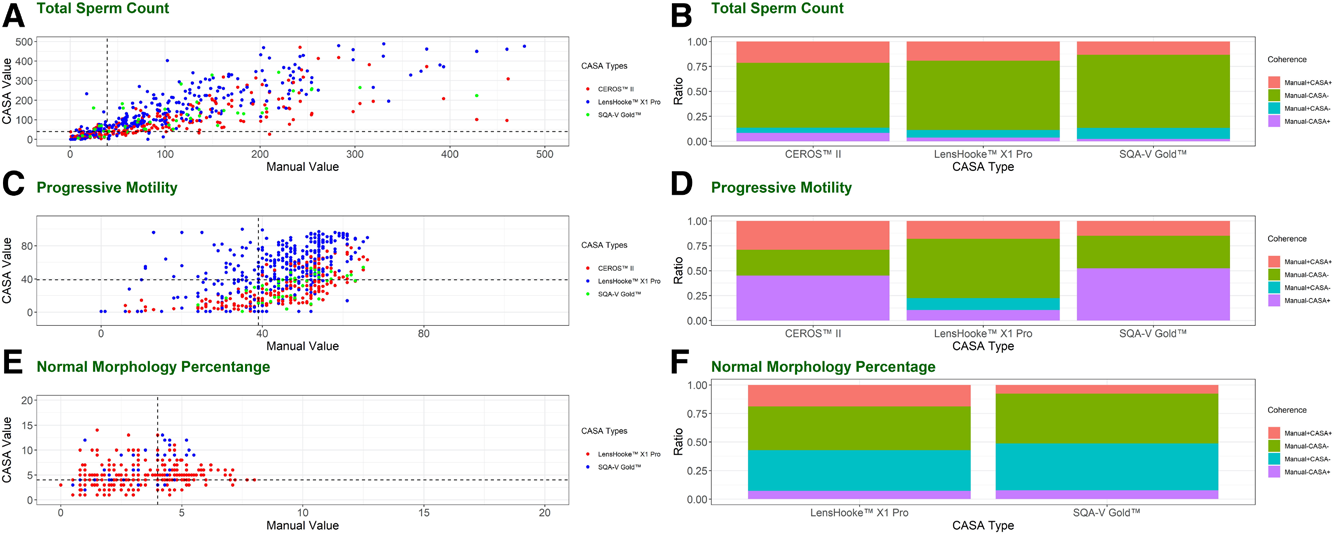
Analysis of oligozoospermia, asthenozoospermia, and teratozoospermia. LensHooke X1 Pro performed the best in oligozoospermia, asthenozoospermia, and teratozoospermia compared with the others. The figure shows the results of the diagnosis comparisons. The dashed lines in (A, C, and E) represented the limits given by the WHO to judge the abnormalities of sperm. (B, D, and F) represented the ratios of the judgment given by manual and different CASA methods. In the legend, the “+” and “−” represented the positive and negative diagnosis results by the corresponding method (e.g. Manual + CASA + meant both the manual method and the CASA method got the same diagnosis.). The total area of pink and green indicates the total accuracy of the diagnostic of patients. CASA = computer-assisted sperm analysis, WHO = World Health Organization.

In the 4 methods, participants considered with oligozoospermia (total sperm count < 39 × 10^6^ per ejaculation), asthenozoospermia (total motility < 40%), and teratozoospermia (normal sperm morphology < 4%) were summarized in Table 3. Participants considered with any of the diseases were different using different methods and the judgments were shown in Figure 3 and Table 4.

**Table 3:** Semen parameter values of participants with oligozoospermia, asthenozoospermia, and teratozoospermia.

Semen parameters		Manual	CEROS II	LensHooke™ X1 Pro	SQA-V Gold™
		Median (5th centiles to 95th centiles)			
Oligozoospermia (total sperm count <39 × 10^6^)	Sample size	86	61	76	7
	Semen volume (mL)	1.95 (0.50–5.08)	1.90 (0.50–4.70)	2.32 (0.73–5.22)	2.3 (1.63–5.03)
	Sperm concentration (×10^6^/mL)	8.60 (0.95–32.90)	9.10 (2.28–36.80)	5.70 (0.09–29.00)	7.7 (1.9–14.5)
	Total sperm count (×10^6^)	16.90 (2.06–36.60)	20.80 (3.84–37.60)	15.90 (0.24–36.90)	14.10 (7.74–31.60)
	Total motility (PR + NP%)	43.00 (11.00–56.80)	15.70 (1.84–51.30)	41.00 (1.00–96.00)	50 (23.40–63.40)
	PR (%)	39.00 (9.00–52.80)	15.60 (1.35–51.00)	33.00 (1.00–96.00)	20.00 (4.40–32.00)
	NP (%)	4.00 (1.00–7.00)	0.19 (0.00–1.47)	1.00 (1.00–29.00)	23.00 (18.60–33.20)
	IM (%)	57.00 (43.20–89.00)	84.30 (48.70–98.20)	59.00 (4.00–100.00)	50.00 (36.60–76.60)
	Normal morphology (%)	2.00 (0.50–4.30)	–	4.00 (1.00–10.00)	5.00 (4.00–6.00)
Asthenozoospermia (PR < 40%)	Sample size	96	122	140	27
	Semen volume (mL)	2.40 (0.80–5.20)	2.4 (0.85–5.05)	2.45 (0.80–5.10)	2.30 (1.23–4.55)
	Sperm concentration (×10^6^/mL)	18.60 (1.07–89.40)	22.70 (3.77–98.40)	33.50 (0.09–157.00)	55.20 (7.88–106.00)
	Total sperm count (×10^6^)	44.40 (2.47–231.00)	51.10 (6.38–310.00)	85.3 (0.29–85.30)	101.00 (14.90–315.00)
	Total motility (PR + NP %)	35 (10.80–43.00)	16.20 (3.36–32.40)	32.50 (1.00–59.10)	41.00 (14.00–60.30)
	PR (%)	31.00 (9.00–39.00)	15.80 (3.36–31.70)	22.00 (1.00–39.00)	19.00 (5.50–38.00)
	NP (%)	4.00 (1.00–7.00)	0.27 (0.00–1.94)	7.00 (1.00–29.00)	12.00 (4.00–28.50)
	IM (%)	65.00 (57.00–89.20)	83.80 (67.60–96.60)	67.50 (40.90–100.00)	59.00 (39.70–86.00)
	Normal morphology (%)	2.00 (0.71–5.13)	–	4.00 (1.00–9.00)	4.00 (3.00–9.00)
Teratozoospermia (normal morphology < 4%)	Sample size	160	–	90	6
	Semen volume (mL)	2.70 (0.90–5.80)	–	2.7 (1.04–5.20)	2.05 (1.50–3.70)
	Sperm concentration (×10^6^/mL)	23.80 (4.30–75.70)	–	28.00 (0.09–184.00)	71.80 (24.60–126.00)
	Total sperm count (×10^6^)	66.20 (11.20–228.00)	–	80.00 (0.26–329.00)	147.00 (62.50–258.00)
	Total motility (PR + NP %)	48.50 (26.00–61.00)	–	53.50 (1.00–86.00)	14.00 (9.50–30.20)
	PR (%)	44.00 (20.00–57.00)	–	39.00 (1.00–73.60)	10.50 (5.25–12.50)
	NP (%)	4.00 (1.97–7.00)	–	9.00 (1.00–32.10)	4.50 (3.25–17.80)
	IM (%)	52.00 (39.00–74.00)	–	46.50 (14.00–100.00)	86.00 (69.80–90.50)
	Normal morphology (%)	2.00 (0.80–3.50)	–	3.00 (1.00–3.00)	3.00 (2.25–3.00)

This table shows the different semen parameter values of patients who suffered from oligozoospermia, asthenozoospermia, and teratozoospermia using manual and 3 CASA methods in medians and ranges from the 5th centiles to the 95th centiles.

CASA = computer-assisted sperm analysis, IM = immotility, NP = nonprogressive motility, PR = progressive motility.

**Table 4:** Judgment of oligozoospermia, asthenozoospermia, and teratozoospermia using different methods.

		CEROS II +	CEROS II −		LensHooke X1 pro +	LensHooke X1 pro −		SQA-V gold +	SQA-V gold −
Oligozoospermia (total sperm count < 39 × 10^6^)	Manual +	44	12	Manual +	62	24	Manual +	6	5
	Manual −	11	134	Manual −	12	225	Manual −	1	33
	κ/*P*-value	0.664	<0.01	κ/*P*-value	0.701	0.001	κ/*P*-value	0.588	0.001
Asthenozoospermia (PR < 40%)	Manual +	60	0	Manual +	73	23	Manual +	6	0
	Manual −	93	53	Manual −	64	162	Manual −	21	13
	κ/*P*-value	0.249	0.001	κ/*P*-value	0.425	0.001	κ/*P*-value	0.157	0.06
Teratozoospermia (normal morphology < 4%)				Manual +	54	102	Manual +	3	16
				Manual −	21	110	Manual −	3	17
				κ/*P*-value	0.177	0.001	κ/*P*-value	0.008	0.946

This table shows the judgment of participants who were considered with the problem of oligozoospermia, asthenozoospermia, and teratozoospermia using the manual method or 1 of the CASA systems.

CASA = computer-assisted sperm analysis.

LensHooke X1 Pro performed the best. It showed substantial performances in oligozoospermia with κ = 0.701 and moderate performances in asthenozoospermia when compared to the manual method with κ = 0.405. CEROS II also showed substantial performances in oligozoospermia (κ = 0.664), while the system had a fair agreement in asthenozoospermia (κ = 0.249). SQA-V Gold had the poorest performance with only moderate agreement in oligozoospermia (κ = 0.588) and even none to slight agreement in asthenozoospermia (κ = 0.157) (Table 4).

**Figure 4.**
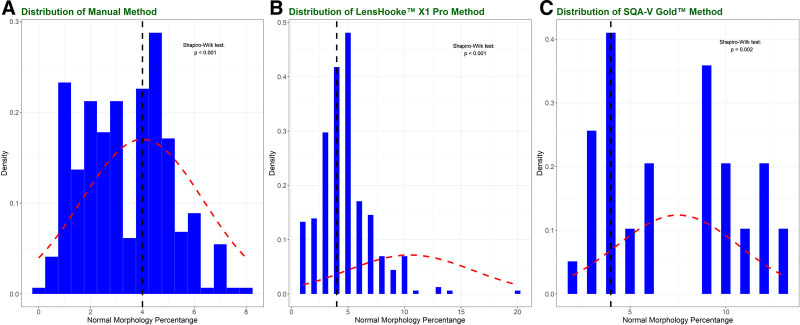
Distribution of the methods of morphology evaluation. When using LensHooke X1 Pro or CEROS II in clinical application, the treatments tended to skew to conventional IVF instead of ICSI. The figure shows the distribution of the morphology results from the 3 methods. The black dotted lines represented the result of 4%, considered as the broad line of teratozoospermia. Proportions to the left of the black dotted lines represented those skewed to ICSI, while the proportions to the right represented those skewed to IVF based on the morphology evaluations. The red-dotted curves represented the normal distributions based on the mean and SD. The results of the test of the normal distribution are shown at the upper right of each figure. ICSI = intracytoplasmic sperm injection, IVF = in vitro fertilization, SD = standard deviation.

Patients whose sperm have a high rate of morphological abnormality tend to use ICSI for effective treatment. None to slight agreements (κ = 0.177) were shown in the diagnosis of morphology using LensHooke X1 Pro (*P*-value = .001) (Table 4), indicating a significant difference between κ of LensHooke X1 Pro and 0. The CASA ICSI ratio was only around 0.31, which was lower than the manual ICSI ratio of 0.54 which was similar to that in the clinical practice of around 0.5, indicating around 2/5 ICSI work would be reduced in the routine treatment. When using SQA-V Gold to evaluate the morphology, even no agreement (κ = 0.008) was shown in the comparison, and the κ of SQA-V Gold showed no significant difference with 0 (*P*-value = .946). In this comparison, the manual ICSI ratio was around 0.49, which was similar to the ratio in the clinical practice, while SQA-V Gold got a much lower result with a ratio of 0.15, indicating around 2/3 ICSI work would be reduced in the routine treatment (Fig. 4).

## 4. Discussion

In this study, we used 3 CASA systems: Hamilton-Thorne CEROS II Clinical, LensHooke™ X1 Pro, and SQA-V Gold™ sperm quality analyzer. To test the accuracy of the CASA systems and evaluate their potential clinical application, we compared the results with manual ones. The parameters compared included concentration, motility, and morphology.

In the concentration evaluation, LensHooke X1 Pro had the best performance among the 3 systems. This might be due to the novel artificial intelligence optical microscopic technology of LensHooke X1 Pro, and the lowest direction limit at 0.1 × 10^6^ sperm/mL compared with the other 2 CASA systems.^[32]^ However, it tended to overestimate the concentration compared with the manual method, as well as the total sperm count. However, the other 2 CASA systems only had moderate performances. In the assessment of sperm motility, only CEROS II had moderate performances but it also tended to underestimate the total motility. The motile and immotile sperm were identified by color-coded tracks and red dots in the CEROS II system respectively, and the interactive illumination check allowed the accurate analysis. Also, the total motility of LensHooke X1 Pro was underestimated, which is consistent with the results in Agarwal et al, 2019.^[33]^ In the evaluation of sperm morphology, LensHooke X1 Pro and SQA-V Gold both could only have poor performances with low consistencies, indicating the current algorithms cannot match the strict rating of the sperm morphology. However, in 1 paper which also focused on the comparison of manual SA and CASAs including LensHooke X1 Pro, the results showed a high level of concordance with 31 semen samples and even showed advantages in accessing the sperm morphology.^[32]^ The different results might result from the processing procedure. 31 semen samples from 13 individuals were divided into 101 aliquots through dilution or concentration, which might reduce the variations caused by debris or other cells and thus increase the correlations. Additionally, a systematic review also pointed out that the low or high concentrations would increase the variability of CASA results, which might also make our results different.^[20]^

In the fertility diagnoses, LensHooke X1 Pro and CEROS II had substantial consistency with manual performances in diagnosing oligozoospermia. Since LensHooke X1 Pro had high consistency in evaluating concentration, it showed moderate agreement in judging oligozoospermia with the manual method. No other stronger agreements could be observed. Among the 3 CASAs, LensHooke X1 Pro performed the best in predicting oligozoospermia, asthenozoospermia, and teratozoospermia. The application of artificial intelligence optical microscopic technology of this CASA contributed to capturing the characteristics of sperm delicately than the conventional CASAs. Thus, when the patients have the likelihood to suffer from 1 of the sperm abnormalities, it is better to conduct SA by andrologists. The lower reliability of the CASA systems seems to give a mistaken report and may delay the treatment.

In our Unit, the ratio of ICSI approximates 0.5 regarding the morphology. However, when using LensHooke X1 Pro or SQA-V Gold to evaluate the morphology, the related ratios were only around 0.31 and 0.15, which both were far lower than the routine standard. There were disagreements in choosing the IVF or ICSI for the patients. If applying CASA in clinical practice, more patients would be allocated in conventional IVF instead of ICSI treatment, which might experience higher risk of fertilization failure. These results provide evidence of the more caution needed in the reproduction treatment and indicate that the tested CASA systems cannot replace the manual method in routine SA.

Additionally, our study also had 4 main limitations. We had different sample sizes for different CASA systems. Only 45 patients were analyzed with SQA-V Gold and the small sample size may cause a decrease in agreement level. The unbalanced sample sizes might cause a loss of power. However, each CASA system was compared with the manual method, which was considered the gold standard, and the comparisons were pairwise. Additionally, none of the patients went through the analyses of sperm morphology using Hamilton-Thorne CEROS II since this system had low accuracy from our previous experience. Additionally, we conducted the bootstrapping analysis based on 1000 replicates to evaluate the uncertainty. The blinding was not performed when allocating the samples into different automated analyses due to the limited resources, which might cause potential observer bias. The values of manual methods were conducted by only 1 andrologist, though the personnel was well trained, and our andrology lab passes the UK NEQAS, which might still cause subjectivity. This study only provided internal comparisons and lacked external validations from other laboratories, which might weaken the generalization of the results. Additionally, the operators have followed the instructions provided by the manufactures and have little information on the machine algorithms. Based on the limitations, in future studies, the sample size imbalance, blinding when allocating samples, operations by multiple andrologists, and the validation across multiple centers should be implemented carefully. Since CASA systems become increasingly important in the reproductive field, the algorithms should be improved further to obtain reproducible results with high consistency, especially in morphology, which has a strict standard. Also, quality control and quality assurance of CASA systems should be taken into consideration.

In this study, the SA results were not consistent when compared to the manual method, and the deviation might result in IVF/ICSI volume changes in the subsequent treatment. Although CASA systems are convinced to have the characteristics of objectivity, faster analytical time, and high reproducibility, they also have disadvantages when compared with the routine manual SA. If applying them in ART clinics, more patients were misrecognized as only needing conventional IVF. Although the technologies used in CASA have been improving recently, our results showed that the manual method could not be replaced by the tested CASA systems at present and the results should be treated with caution.

## 5. Conclusion

CASA results were not consistent with manual results, and the deviations might result in skewed IVF/ICSI allocation in subsequent treatment. Interestingly, tested CASA systems tend to skew to conventional IVF instead of ICSI. Although CASA technologies have been improving recently, the manual method cannot be replaced by the tested CASA systems at present and the results should be treated with caution. CASA algorithms should be improved, especially in morphology. Future strict studies should be designed to evaluate the CASA systems with both internal and external validations.

## Acknowledgments

The work was supported by Right Pearl Limited (TR1914524).

## Author contributions

**Conceptualization:** David Yiu Leung Chan.

**Data curation:** Murong Xu, Mingpeng Zhao, Carol Pui Shan Chan, Ka Kei Fung.

**Formal analysis:** Murong Xu, Mingpeng Zhao, Huixia Yang, Minqi Liu.

**Investigation:** Murong Xu, Mingpeng Zhao, Huixia Yang, Minqi Liu, Carol Pui Shan Chan, Ka Kei Fung.

**Methodology:** Murong Xu, Carol Pui Shan Chan, Ka Kei Fung.

**Resources:** Jacqueline Pui Wah Chung, Ellis Kin Lam Fok, David Yiu Leung Chan.

**Software:** Murong Xu, Mingpeng Zhao.

**Supervision:** Jacqueline Pui Wah Chung, Ellis Kin Lam Fok, David Yiu Leung Chan.

**Visualization:** Murong Xu, Mingpeng Zhao, David Yiu Leung Chan.

**Writing – original draft:** Murong Xu, Mingpeng Zhao.

**Writing – review & editing:** Murong Xu, Mingpeng Zhao, David Yiu Leung Chan.

## References

[1] Agarwal A Mulgund A Hamada A Chyatte MR. A unique view on male infertility around the globe. Reprod Biol Endocrinol. 2015;13:1–9. 25928197 10.1186/s12958-015-0032-1 PMC4424520

[2] Mascarenhas MN Flaxman SR Boerma T Vanderpoel S Stevens GA. National, regional, and global trends in infertility prevalence since 1990: a systematic analysis of 277 health surveys. PLoS Med. 2012;9:e1001356. 23271957 10.1371/journal.pmed.1001356 PMC3525527

[3] Kumar N Singh AK. Trends of male factor infertility, an important cause of infertility: a review of literature. J Hum Reprod Sci. 2015;8:191–6. 26752853 10.4103/0974-1208.170370 PMC4691969

[4] Van Balen F Gerrits T. Quality of infertility care in poor-resource areas and the introduction of new reproductive technologies. Hum Reprod. 2001;16:215–9. 11157809 10.1093/humrep/16.2.215

[5] Engel KM Grunewald S Schiller J Paasch U. Automated semen analysis by SQA vision® versus the manual approach: a prospective double-blind study. Andrologia. 2019;51:e13149. 30255510 10.1111/and.13149

[6] Baskaran S Finelli R Agarwal A Henkel R. Diagnostic value of routine semen analysis in clinical andrology. Andrologia. 2021;53:e13614. 32400107 10.1111/and.13614

[7] Agarwal A Gupta S Sharma R. Andrological Evaluation of Male Infertility. Cham: Springer; 2016:113–133.

[8] Tomlinson M. Uncertainty of measurement and clinical value of semen analysis: has standardisation through professional guidelines helped or hindered progress? Andrology. 2016;4:763–70. 27529487 10.1111/andr.12209

[9] Punjabi U Wyns C Mahmoud A Vernelen K China B Verheyen G. Fifteen years of Belgian experience with external quality assessment of semen analysis. Andrology. 2016;4:1084–93. 27410398 10.1111/andr.12230

[10] Franken DR Aneck-Hahn N Lombaard C Kruger TF. Semenology training programs: 8 years’ experience. Fertil Steril. 2010;94:2615–9. 20553675 10.1016/j.fertnstert.2010.04.048

[11] Agarwal A Sharma R Gupta S. Standardized laboratory procedures, quality control and quality assurance are key requirements for accurate semen analysis in the evaluation of infertile male. World J Mens Health. 2022;40:52–65. 33987999 10.5534/wjmh.210022 PMC8761242

[12] Group EASI. Guidelines on the application of CASA technology in the analysis of spermatozoa. Hum Reprod. 1998;13:142–5. 9512246

[13] Amann RP Waberski D. Computer-assisted sperm analysis (CASA): capabilities and potential developments. Theriogenology. 2014;81:5–17.e1. 24274405 10.1016/j.theriogenology.2013.09.004

[14] Agarwal A Said TM. Interpretation of basic semen analysis and advanced semen testing. Male Infertility Probl Solut. 2011:15–22.

[15] Schubert B Badiou M Force A. Computer-aided sperm analysis, the new key player in routine sperm assessment. Andrologia. 2019;51:e13417. 31475742 10.1111/and.13417

[16] Ehlers J Behr M Bollwein H Beyerbach M Waberski D. Standardization of computer-assisted semen analysis using an e-learning application. Theriogenology. 2011;76:448–54. 21529919 10.1016/j.theriogenology.2011.02.021

[17] Zhu J Pacey A Barratt C Cooke I. Computer-assisted measurement of hyperactivation in human spermatozoa: differences between European and American versions of the Hamilton-Thorn motility analyser. Hum Reprod. 1994;9:456–62. 8006134 10.1093/oxfordjournals.humrep.a138527

[18] Yeung CH Nieschlag E. Performance and comparison of CASA systems equipped with different phase-contrast optics. J Androl. 1993;14:222–8. 8407578

[19] Liu D Baker H. Evaluation and assessment of semen for IVF/ICSI. Asian J Androl. 2002;4:281–6. 12508129

[20] Finelli R Leisegang K Tumallapalli S Henkel R Agarwal A. The validity and reliability of computer-aided semen analyzers in performing semen analysis: a systematic review. Transl Androl Urol. 2021;10:3069–79. 34430409 10.21037/tau-21-276 PMC8350227

[21] Lammers J Splingart C Barrière P Jean M Fréour T. Double-blind prospective study comparing two automated sperm analyzers versus manual semen assessment. J Assist Reprod Genet. 2014;31:35–43. 24242989 10.1007/s10815-013-0139-2 PMC3909144

[22] Talarczyk-Desole J Berger A Taszarek-Hauke G Hauke J Pawelczyk L Jedrzejczak P. Manual vs. computer-assisted sperm analysis: can CASA replace manual assessment of human semen in clinical practice? Ginekol Pol. 2017;88:56–60. 28326513 10.5603/GP.a2017.0012

[23] Organization WH. WHO laboratory manual for the examination and processing of human semen. 2010. 21243747

[24] Bartko JJ. The intraclass correlation coefficient as a measure of reliability. Psychol Rep. 1966;19:3–11. 5942109 10.2466/pr0.1966.19.1.3

[25] Koo TK Li MY. A guideline of selecting and reporting intraclass correlation coefficients for reliability research. J Chiropr Med. 2016;15:155–63. 27330520 10.1016/j.jcm.2016.02.012 PMC4913118

[26] Peng C-YJ Lee KL Ingersoll GM. An introduction to logistic regression analysis and reporting. J Educ Res. 2002;96:3–14.

[27] Cohen J Cohen P West SG Aiken LS. Applied Multiple Regression/Correlation Analysis for the Behavioral Sciences. Routledge; 2013.

[28] Doğan NO. Bland-Altman analysis: a paradigm to understand correlation and agreement. Turk J Emerg Med. 2018;18:139–41. 30533555 10.1016/j.tjem.2018.09.001 PMC6261099

[29] Myles PS Cui J. I. Using the Bland–Altman Method To Measure Agreement With Repeated Measures. Oxford University Press; 2007. 10.1093/bja/aem214 17702826

[30] McHugh ML. Interrater reliability: the kappa statistic. Biochem Med (Zagreb). 2012;22:276–82. 23092060 PMC3900052

[31] DiCiccio TJ Efron B. Bootstrap confidence intervals. Stat Sci. 1996;11:189–228.

[32] Agarwal A Selvam MKP Ambar RF. Validation of LensHooke® X1 PRO and computer-assisted semen analyzer compared with laboratory-based manual semen analysis. World J Mens Health. 2021;39:496. 33663026 10.5534/wjmh.200185 PMC8255407

[33] Agarwal A Henkel R Huang CC Lee MS. Automation of human semen analysis using a novel artificial intelligence optical microscopic technology. Andrologia. 2019;51:e13440. 31583732 10.1111/and.13440

